# Structural and effective connectivity reveals potential network-based influences on category-sensitive visual areas

**DOI:** 10.3389/fnhum.2015.00253

**Published:** 2015-05-07

**Authors:** Nicholas Furl

**Affiliations:** ^1^MRC Cognition and Brain Sciences UnitCambridge, UK; ^2^Department of Psychology, Royal Holloway, University of LondonEgham, UK

**Keywords:** fusiform face area, face perception, dynamic causal modelling, diffusion tensor imaging, effective connectivity, visual cortex, lateral occipital complex, functional integration

## Abstract

Visual category perception is thought to depend on brain areas that respond specifically when certain categories are viewed. These category-sensitive areas are often assumed to be “modules” (with some degree of processing autonomy) and to act predominantly on feedforward visual input. This modular view can be complemented by a view that treats brain areas as elements within more complex networks and as influenced by network properties. This network-oriented viewpoint is emerging from studies using either diffusion tensor imaging to map structural connections or effective connectivity analyses to measure how their functional responses influence each other. This literature motivates several hypotheses that predict category-sensitive activity based on network properties. Large, long-range fiber bundles such as inferior fronto-occipital, arcuate and inferior longitudinal fasciculi are associated with behavioral recognition and could play crucial roles in conveying backward influences on visual cortex from anterior temporal and frontal areas. Such backward influences could support top-down functions such as visual search and emotion-based visual modulation. Within visual cortex itself, areas sensitive to different categories appear well-connected (e.g., face areas connect to object- and motion sensitive areas) and their responses can be predicted by backward modulation. Evidence supporting these propositions remains incomplete and underscores the need for better integration of DTI and functional imaging.

A current challenge for visual neuroscience is to explain how perception of complex and meaningful objects is achieved. High-level vision is associated with anatomically-focal, functionally-defined brain areas in human occipitotemporal cortex, usually localized using functional magnetic resonance imaging (fMRI). These “category-sensitive” areas are typically inferred to be specialized for processing their preferred visual categories (i.e., they are domain specific or modular) (Kanwisher, 2000). Experimental manipulation of anatomically-localized responses—functional segregation—is a prevalent technique for brain imaging research. However, research need not exclusively focus on anatomically-localized processing. Functional integration (Friston and Price, 2011), emphasizes network-level explanations for high-level visual function (Ishai, 2008; Wiggett and Downing, 2008; Atkinson and Adolphs, 2011). Functional integration and functional segregation are complementary approaches and a comprehensive research program should pursue both.

The present article reviews studies taking a functional integration approach. These studies either map structural anatomic connections between brain areas using diffusion tensor imaging (DTI) or estimate their effective connectivity using functional brain imaging methods including fMRI, electroencephalography (EEG) or magnetoencephalography (MEG). However, few studies to date have combined DTI and effective connectivity techniques. DTI maps structural connections composed of axonal fiber tracts. In contrast, effective connectivity measures how functional responses influence (or “cause”) each other. However, they share the similar goal of inferring functional integration, with DTI showing which areas are connected and effective connectivity showing the experimental conditions under which these structural connections might enact their influence. Studies using these methods have not provided a comprehensive picture of human structural connections or the effective interactions supported by these structures. Nevertheless, the findings reviewed here suggest specific structural and effective network properties that are relevant to visual analysis of complex stimuli. These network properties include influences of major, long-range fiber bundles, backward connections from higher to lower-level areas and interactions between areas with dissimilar category preferences. These network properties can potentially explain diverse phenomena, including top-down, attentional and emotional modulation of visual cortex, oscillatory power changes and repetition-related response modulations. This review covers these findings, discusses the deficiencies of existing theories and describes how a functional integration approach is well-suited to advance understanding of visual processing in human occipitotemporal cortex.

## Hierarchical Connectivity Influences Functional Responses

Responses in category-sensitive brain areas are likely to be influenced by the different types of connections associated with hierarchical networks. Hierarchical structures (Felleman and Van Essen, 1991) distinguish connections based on forward, backward, horizontal and long-range “shortcut” contributions. Much of the work distinguishing these hierarchical influences is based on physiological and computational methods. Alternatively, DTI and effective connectivity are suitable methods for identifying and distinguishing influences of these different types of connections* in vivo* in humans.

In hierarchically-connected networks, stimuli are assumed to initially drive a cascade of responses up successive levels of the hierarchy. This bottom-up or stimulus-driven “feedforward sweep” appears sufficient to generate many selective low-level visual responses, most notably the spatial receptive field properties of cells in retinotopic visual cortex (Maunsell and Newsome, 1987). The first 100–150 ms of responses are often attributed to this “feedforward sweep” (Lamme and Roelfsema, 2000), although at least local horizontal and backward connections might also influence even these early responses in the visual system (DiCarlo and Cox, 2007). Intracranial recording in monkeys and humans have attempted to isolate feedforward influences by measuring the first spikes evoked by visual stimuli. The first spikes can be sensitive to object categories, including faces (Oram and Perrett, 1992; Tovée, 1994; Liu et al., 2009). Indeed, rank-order codes based on the first spike in feedforward networks can explain fast perceptual responses. In contrast to firing rate codes, which must be integrated over temporal durations (VanRullen et al., 2005), rank-order codes can be decoded more quickly. High-level category response preferences generated solely from a feedforward sweep are also predicted by a variety of feedforward computational models (Riesenhuber and Poggio, 1999; Giese and Poggio, 2003; Lange and Lappe, 2006; Serre et al., 2007; Masquelier et al., 2011). Given the capabilities of feedforward networks, it is pertinent to understand the extent to which feedforward communication in the brain generates category-sensitive responses in practice.

However, the feedforward sweep does not easily explain all high-level category preferences. Backward influences might explain, for example, extra-classical receptive field effects (Rao and Ballard, 1999; Hosoya et al., 2005), attention (Corbetta and Shulman, 2002) and how early (perhaps feedforward) responses to faces are modulated at later times by facial identity or expression (Sugase et al., 1999). Computational models also assign an essential role to backward connections. In the case of predictive coding models, hierarchical structures encode the causes of external stimuli by transmitting predictions via backward connections and their errors via forward connections. Perception and learning occur when top-down predictions are adjusted by the bottom-up prediction error until the prediction error is minimized (Rao and Ballard, 1999; Bastos et al., 2012). Below, this review describes data on repetition suppression (Ewbank et al., 2011, 2013) and non-linear oscillatory modulation (Chen et al., 2009; Bastos et al., 2012) that is consistent with this hypothetical role of backwards connections in predictive coding.

Forward and backward connections need not be restricted to connecting anatomically- or hierarchically-adjacent levels. Shortcut and long-range connections allow information to skip levels. Expedited access of these higher level areas may trigger earlier top-down influences of backward connections, before the feedforward sweep completes. Some authors have hypothesized shortcut connections. For example, a shortcut pathway could trigger early perceptual predictions in orbitofrontal cortex (OFC), which would then guide visual object processing via backward connections (Bar, 2003; Kveraga et al., 2007). Similarly, emotion sensitivity in visual areas might arise when a shortcut pathway initially activates the amygdala. Expedited assessment of emotion-related information by the amygdala could then quickly modulate visual responses in occipitotemporal cortex via backward connections (Morris et al., 1998, 1999; Pessoa and Adolphs, 2010; Furl et al., 2013).

Visual cortex appears to contain a hierarchical apparatus and therefore response specificity in visual cortex likely depends on distinguishable forward and backward influences. Moreover, mainstream computational models make important assumptions about the influences of forward and backward influences. While many models rely solely on forward influences, models such as predictive coding posit specific roles for backward connections. While the DTI and effective connectivity studies performed to date and reviewed herein do not yet support any particular computational theory in detail, they provide evidence for a role of backward influences during perception of high-level visual stimuli.

## Is there Hierarchical Organization between Areas Sensitive to the Same Category?

Multiple areas that are sensitive to the same category have been described as hierarchies. At the most general level in visual cortex, all areas may be associated with the dorsal or ventral hierarchical streams (Mishkin and Ungerleider, 1982; Goodale and Milner, 1992), which are dedicated to spatial representation for visually-guided actions (dorsal stream) and for object recognition (ventral stream). Many category-sensitive areas appear associated with the ventral stream, including the well-studied fusiform face area (FFA) in the fusiform gyrus (Kanwisher et al., 1997). FFA responds more to faces than non-face objects, as do areas in the ventral occipital lobe (OFA) and posterior superior temporal sulcus (STS). An influential network-based theory for face perception (Haxby et al., 2000) links these three face-sensitive areas into a “core” system for visual analysis of faces. OFA (at a lower-hierarchical level) processes facial features and FFA uses these features to process facial identity, while posterior STS independently processes changeable aspects of faces (expression, eye gaze, etc.). These so-called core areas are responsible for visual analysis of faces and they feed their outputs forward to extended areas including the amygdala, insula and frontal cortex. Although Haxby et al. explicitly emphasizes the functions of feedforward interactions, there is room also for backward interactions with the extended system. For example, the amygdala, together with occipitotemporal areas, show enhanced responses to fearful faces (Sabatinelli et al., 2011), a phenomenon that may be due to the influence of backwards connectivity from the amygdala to visual cortex (Furl et al., 2013). The main features of the Haxby et al. (2000) framework remain an organizing principle for face perception research (Haxby and Gobbini, 2011), although updates have been proposed (O’Toole et al., 2002; Calder and Young, 2005; Atkinson and Adolphs, 2011; Calder, 2011; Haxby and Gobbini, 2011; Wieser and Brosch, 2012).

There are many areas sensitive to categories other than faces. Dorsal occipitotemporal areas show preferences for body actions and biological motion perception (Giese and Poggio, 2003; Kilner, 2011) including hMT+/V5 and an area in posterior STS (Peuskens et al., 2005; Grosbras et al., 2012). This posterior STS area resembles the face-sensitive area in posterior STS (Hein and Knight, 2008), which may also reflect representations of facial actions (O’Toole et al., 2002; Calder and Young, 2005; Haxby and Gobbini, 2011). Computational models of biological motion are typically hierarchical (Giese and Poggio, 2003; Lange and Lappe, 2006) and demonstrate how purely feedforward connections could give rise to these responses in posterior STS. There are also responses sensitive to biological motion outside of occipitotemporal cortex, including responses in inferior frontal cortex in the vicinity of premotor cortex (Saygin et al., 2004) that may be associated with the mirror neuron system. Indeed, a theory based on hierarchical predictive coding has been proposed for the biological motion system, where responses in the STS to visually-presented actions are hypothesized to depend crucially on backward connections from the mirror neuron system (Kilner, 2011). And, there is also biological motion sensitivity in the cerebellum (Sokolov et al., 2012), where lesions produce deficits in biological motion perception (Sokolov et al., 2010).

Other areas show response preferences for scenes, word forms, objects or bodies. There are at least three areas sensitive to scenes (Nasr et al., 2011) including areas in the transverse occipital sulcus (TOS), retrosplenial cortex (RSC) and parahippocampal gyrus (PPA). There is also a putative visual word form system in left occipitotemporal cortex (Dehaene and Cohen, 2011), which appears to be divided into hierarchically-organized sub-areas, which respond to progressively more complex word forms along a posterior to anterior gradient (Vinckier et al., 2007). A posterior to anterior hierarchical organization has also been proposed for category-sensitive areas that appear in pairs (Taylor and Downing, 2011). Sensitivity to intact vs. scrambled objects is observed in the lateral occipital complex (LOC). LOC, thought to be specialized for object processing, appears to be divided into posterior (LO) and an anterior subregions (pFs) (Grill-Spector et al., 1999; Haushofer et al., 2008). Similarly, sensitivity to bodies vs. objects is divided into posterior extrastriate body (EBA) and anterior fusiform body (FBA) areas (Peelen and Downing, 2007).

To summarize, several visual categories are associated with selective responses in small numbers of occiptotemporal areas. Many authors have proposed hierarchical organizations for these areas that either do not define a detailed distinction between forward and backward connectivity (Taylor and Downing, 2011) or emphasize predominantly forward connectivity (Haxby et al., 2000; Giese and Poggio, 2003; Lange and Lappe, 2006). The Haxby et al. (2000) theory is an example of a theory that assigns visual analysis functions only to occipitotemporal areas sensitive to the same category (faces), with little role described for other relevant occipitotemporal visual areas (LOC or hMT+/V5).

## DTI and Effective Connectivity Methods

The following sections rely on results using DTI and effectively connectivity methods. Before proceeding, this section briefly reviews these methods, including some of their strengths and weaknesses. Structural connectivity studies generally report deterministic or probabilistic tractography of DTI data, which reveal the anatomic paths over which axonal fibers travel. Tractography cannot measure the direction of information transmission or whether any tract is a forward or backward connection. Fiber pathways provide the physical means by which different areas can influence each other’s responses (effective connectivity). Effective and structural connectivity measures are complementary, even though their relationship is not fully understood (Woolrich and Stephan, 2013). While structural connections enable effective connectivity, plasticity can alter the ability of a physical structure to transmit information and this plasticity may be captured by effective connectivity measures. Stephan et al. (2009b) have suggested that knowledge about structural connectivity can be implemented as a Bayesian prior on effective connectivity model solutions (Stephan et al., 2009b).

For effective connectivity, most studies of category sensitivity have used dynamical causal modeling (DCM; Friston et al., 2003). DCM implements a generative model with three types of connectivity-based parameters estimated from imaging data. Exogenous inputs measure how much stimulus presentation perturbs the model’s activity dynamics. Endogenous parameters measure connectivity that is consistent across experimental conditions. Bilinear parameters measure connectivity that is modulated by an experimental manipulation and are typically of most interest. For example, a category-specific modulation of a directed connection can demonstrate that one area “causes” its connected area to have a category-sensitive response. These measures are “causal” in the sense that they are directed, where the influence of an area on another can be inferred. These directed measures are useful, for example, for testing hypotheses about the roles of forward vs. backward connections.

Early studies of category-sensitive areas specified a fully-connected DCM, estimated its parameters, and then used general linear models to evaluate the parameter significance for each connection (Mechelli et al., 2003, 2004; Summerfield et al., 2006; Rotshtein et al., 2007). In contrast, more recent studies specified a set hypothetical connectivity architectures (the model space) and then used Bayesian model comparison to identify the most likely (optimal) model with the highest model evidence (Penny et al., 2004a, 2010; Stephan et al., 2009a). Selecting an optimal model using model comparison allows both hypothesis testing about network architecture and more accurate parameter estimation (Stephan et al., 2010).

Other effective connectivity methods include structural equation modeling (Büchel and Friston, 1997), path analysis (Lim et al., 2009) and Granger causality analysis. These methods, like DCM, estimate directed coupling. Granger causality (Granger, 1969) predicts a target area’s responses from the recent history of responses in a source area (See Seth, 2010 for one computational implementation). Methods related to Granger causality include partial directed coherence (Baccala and Sameshima, 2001) and transfer entropy (Vakorin et al., 2011). Many studies employ functional connectivity, a notion distinct from effective connectivity. Functional connectivity describes correlations over time between responses in different brain areas and is are not causal because correlations cannot specify the direction of coupling. This review focuses on causal inferences based on effective connectivity analyses.

## Major Fiber Bundles may be Functionally Important for High-Level Vision

One research goal promoted in this review seeks to understand local responses and representations in high-level visual areas by studying the structural “wiring diagram” of brain connections. The most obvious connectivity features in the brain are several large, long-range fiber bundles (fasciculi), mostly oriented along a posterior-anterior axis. These bundles have been observed using human post-mortem dissection and can be imaged using DTI (ffytche and Catani, 2005; Martino et al., 2011; Forkel et al., 2014). The size of these fasciculi are surprising, as the long-range structural connections that they contain are relatively rare. Instead, the brain is dominated by short, local connections (Buzsáki et al., 2004; Bassett et al., 2009). This so-called “small world” organization (favoring short range connections over long range) optimizes wiring efficiency. Thus, given the apparent wiring cost associated with these large, long-range fasciculi, it is of interest to discover what functionality is gained by their presence. One possibility is that they may enable some types of hierarchical processing.

Hierarchical processing in higher-level vision is most likely to be influenced by the inferior longitudinal (ILF), arcuate/superior longitudinal (AF/SLF) and inferior occipitofrontal (IFOF) fasciculi. ILF projects down the length of the temporal lobe, with fibers terminating posteriorly in inferior occipital cortex (near OFA) and fusiform gyrus (near FFA) and anteriorly in the temporal pole, amygdala and hippocampus (Catani et al., 2003). ILF is well-suited to subserve hierarchical processing for the ventral (object recognition) stream. Lateral ILF contains short, U-shaped fibers, which can mediate local processing between successive hierarchical levels. These U-shaped fibers have been denoted the occipito-temporal projection system (Tusa and Ungerleider, 1985). In contrast, medial ILF has long fibers that connect posterior occipitotemporal with anterior temporal areas. These long fibers could operate a fast, feedforward shortcut to anterior areas such as the amygdala. Indeed, it has been proposed that the amygdala can modulate visual responses via backward connections in the presence of emotional stimuli (Morris et al., 1998, 1999; Vuilleumier and Pourtois, 2007; Furl et al., 2013).

Other large fiber bundles connect occipitotemporal areas with frontal cortex. The arcuate (AF) and superior longitudinal (SLF) fasciculi share overlapping anatomical courses and are often considered together (Martino et al., 2011). These tracts innervate temporal cortex, curve around the temporal parietal junction and then project towards inferior frontal cortex. Like ILF, AF/SLF is comprised of both long fibers and short, U-shaped fibers (ffytche and Catani, 2005). In the left hemisphere, AF/SLF is considered to subserve language, as it connects specialized language areas in posterior superior temporal lobe and inferior frontal gyrus (Dick and Tremblay, 2012). The functions of the right hemisphere AF/SLF have been less-studied but could be associated with posterior STS (Blank et al., 2011; Ethofer et al., 2011).

Like AF/SLF, IFOF contains long fibers that could provide a shortcut connecting occipitotemporal visual areas with frontal regions, including OFC and the overlapping ventromedial frontal cortex (VMFC). The OFC/VMFC could influence visual areas directly, via IFOF, or indirectly, via the uncinate fasciculus, which connects OFC/VMFC to the anterior temporal lobe and the amygdala (Catani et al., 2002). A number of effective connectivity studies have proposed that OFC or VMFC exert top-down effects on visual cortex, especially during visual search (See section: Backward influences from frontal areas) and the IFOF could convey such a long-range backward influence.

## DTI Connectivity Relates to Recognition Ability

These fiber bundles are structurally well-situated to mediate hierarchical processing for the visual system, and emerging evidence suggests that their properties also relate to behavioral recognition performance. Thomas et al. (2008) studied face perception declines due to aging. Behaviorally, they found age-related declines for discriminations between faces but not cars. Using DTI, Thomas et al. extracted microstructural measures from the major fiber bundles ILF and IFOF, including percentages of fibers and numbers of voxels occupied by each tract as well as fractional anisotropy (FA), a measure of the magnitude of fiber directionality. The selective aging effects on face discrimination performance correlated with declines in IFOF microstructure. Using similar methods, Thomas et al. (2009) compared DTI scans from six healthy individuals exhibiting chronically poor face recognition performance (congenital prosopagnosics) with control individuals exhibiting normal face recognition ability. Deficiencies in congenital prosopagnosic microstructure were observed in ILF, IFOF and the forceps major (which connects occipital areas to the corpus collosum). These data suggest that information communicated by ILF and IFOF contribute to face recognition performance.

More recent studies have, however, produced a more complicated story. Gomez et al. (2015) examined the ILF as well as DTI tracts connected to the fMRI-localized FFA. Gomez et al. showed that the FFA was associated with a large posterior/anterior oriented fiber bundle found ventral to the ILF (as conventionally defined). However, it was in white matter local to the FFA that Gomez found reduced microstructure associated with congenital prosopagnosia and with individual differences in the recognition ability of typical participants. Two further studies, using samples of typical participants but not congenital prosopagnosics (Postans et al., 2014; Tavor et al., 2014) and conventional assessment of the ILF (without using fMRI functional localization), linked individual differences in facial recognition ability to FA in the ILF.

These three studies (Postans et al., 2014; Tavor et al., 2014; Gomez et al., 2015) also examined DTI associations with scene recognition, but found disparate results. Gomez et al. (2015) showed that scene-sensitive cortex was associated with its own fiber bundle and that FA local to scene-sensitive cortex was correlated with scene recognition ability in typical participants. Tavor et al. (2014), however, found scene recognition ability was associated with FA in middle and posterior ILF. Lastly, Postans et al. (2014) found correlations between scene recognition ability and the fornix.

These studies aimed to dissociate different functions (faces, cars or scenes) in different fiber bundles. To date, the details of these dissociations are still not clear, as they are different in each study, with mixed evidence for a selective role of the ILF for face recognition. Although no major fiber bundle is likely to contribute to recognition of any single category (such as face or scene perception), the function of a given fiber bundle might contribute more to recognition of some categories than others. Thus, linking behavioral recognition performance to fiber bundle properties is a promising research method. Research linking fiber bundles with behavior has also been well-served by combining DTI with fMRI functional localization (Gomez et al., 2015). Such studies can more directly link local and long0range connectivity structure with category-sensitive cortex (Gomez et al., 2015).

## Structural Connectivity between Face and Non-Face Sensitive Areas

Combining DTI with fMRI reveals the relationships between structural connectivity and functional responses. New analysis methods show that areas with a certain category preference have identifiable patterns of connectivity with the rest of the brain. That is, an area’s functional response may be determined by which areas connect to it structurally. Saygin et al. (2011) identified the “connectivity fingerprint” of the FFA by using multiple regressions to linearly relate fMRI-measured face sensitivity in each voxel in the fusiform gyrus (where FFA is located) with probabilistic tractography measures to anatomical parcellations throughout the brain. These regressions could then make predictions about the amount of face selectivity when provided patterns of structural connectivity over the whole brain. This model predicted the fMRI data better than multiple control models, including one based on voxel distance from the FFA, demonstrating that structural connectivity was more widespread than in nearby occipitotemporal areas. A follow-up study (Osher et al., 2015) expanded this approach successfully to voxels in the whole brain (not just fusiform gyrus) and to faces, bodies, scenes and objects. They also used several methods, including graph theory, to establish positive relationships between the category-sensitivity of a voxel and the category sensitivity in the patterns of brain parcels most contributing to its structural connectivity fingerprint.

The results of Osher et al. (2015) suggest that parcels whose structural connectivity best predicts a voxel’s category-sensitivity tend to also contain category-sensitive voxels. Nevertheless, there is empirical evidence showing that an area sensitive to a category might also be influenced by areas with different response preferences. Meanwhile, structural connectivity is not always abundant between areas with the same category/preference. These findings are of interest because many studies using conventional functional imaging methods focus only on regions of interest sensitive to the same category as the stimuli (e.g., experiments on faces study only face-sensitive regions of interest). Similarly, theories such as Haxby et al. (2000) describe visual analysis of faces in terms of areas that are sensitive to faces (OFA, FFA, STS).

The FFA, for example, is well-connected to motion and object sensitive occipitotemporal areas. Kim et al. (2006) showed that occipitotemporal tracts were segregated into two pathways. One pathway connected V1, V2, VP and V4v with PPA. The second pathway connected V3 and V3a to a loop including hMT+/V5 (which is motion sensitive), LOC (which is object-sensitive) and FFA (which is face-sensitive). The finding of connections between fusiform gyrus (containing FFA) and the motion-sensitive hMT+/V5 has been replicated. Ethofer et al. (2011) localized areas in the biological motion pathway by comparing fMRI responses to dynamic vs. static gaze shifts and reported the brain areas structurally connected to these biological motion areas. These areas (posterior STS, hMT+/V5, inferior frontal gyrus and insula) were highly interconnected with each other, with the tracts following the course of AF/SLF. hMT+/V5, in particular, was well-connected with ventral occipitotemporal cortex (in the vicinity of OFA and FFA).

Ethofer et al. (2011) also found minimal connectivity between ventral occipitotemporal cortex and posterior STS. This is surprising, since posterior STS and ventral occipitotemporal cortex all contain face-sensitive areas and may constitute a core system for the visual analysis of faces (Haxby et al., 2000). This finding—that posterior STS is weakly connected with ventral occipitotemporal areas—has been replicated multiple times. Gschwind et al. (2012) found that fibers from posterior STS were associated with AF/SLF and inferior frontal gyrus, while low-level visual cortex, OFA, FFA and the amygdala were associated instead with ILF. In another replication, Pyles et al. (2013) found connectivity between FFA, OFA and anterior temporal cortex, with near-zero connectivity between these areas and posterior STS. Lastly, Blank et al. (2011) found that FFA was well-connected with anterior STS but not with the more posterior aspect of STS, where face-selectivity is typically localized.

In summary, structural connectivity findings for face perception suggest a more complicated network that that expected from existing theory (Haxby et al., 2000). FFA appears associated with the ILF (Gschwind et al., 2012) and areas that are not face-sensitive (Kim et al., 2006; Gschwind et al., 2012) such as hMT+/V5 (motion sensitive) and LOC (object-sensitive). Even though posterior STS also contains a face-sensitive area, there is little evidence for direct connectivity between posterior STS and OFA or FFA (Blank et al., 2011; Ethofer et al., 2011; Gschwind et al., 2012; Pyles et al., 2013). Posterior STS also connects to hMT+/V5, and is associated with the AF/SLF (instead of ILF) and inferior frontal gyrus (Ethofer et al., 2011). A modification of Haxby et al. (2000), such as the O’Toole et al. (2002) framework, may be more accurate, with occipital cortex (perhaps early visual areas or OFA) communicating with posterior STS via hMT+/V5. It is not clear yet what information might be transferred between FFA and hMT+/V5, LOC and other connected areas or how this information transfer may relate to behavior.

## Evidence for Backward Influences in Occipitotemporal Cortex

Effective connectivity analysis of functional imaging data is more useful than DTI for addressing questions about directionality of information flow, such as dissociating the roles of forward vs. backward connections. Collectively, the evidence reviewed in this section suggests that backward connections within visual cortex can influence category-sensitive responses in addition to feedforward influences. Some of this evidence, including evidence from studies on repetition suppression using fMRI and neural oscillations using MEG, is consistent with a role for backward connections in predictive coding.

DCM studies sometimes assume*a priori* that occipitotemporal networks have reciprocal forward and backward endogenous connections (interactions between areas that are constant across experimental conditions). These studies therefore only considered reciprocal models (Mechelli et al., 2003, 2004; Summerfield et al., 2006; Rotshtein et al., 2007; Chen et al., 2009; Herrington et al., 2011; Nagy et al., 2012; but see Cohen Kadosh et al., 2011 for a paper where only feedforward models are considered) However, as discussed above, intra-cranial electrophysiological studies and many computational models claim that fast category-sensitive responses might reflect the feedforward sweep alone. For this reason, some DCM studies compared models with endogenous feedforward against those with reciprocal connectivity. Although the prevailing model identified in these studies is reciprocal (Rudrauf et al., 2008; Ewbank et al., 2011, 2013; Liu et al., 2011; Nagy et al., 2012; Goulden et al., 2012; Furl et al., 2013, 2014b), there are exceptions reporting feedforward only models as optimal (Fairhall and Ishai, 2007; Li et al., 2010; Furl et al., 2014a).

It is tempting to relate this minority of studies to simple tasks involving fast processing that may be performed with only a feedforward sweep (Liu et al., 2009) or otherwise relatively small impact of backward connections, as predicted by some computational models (Serre et al., 2007). Future model comparisons should take care to investigate the involvement and measurement of backward connections more fully. Here, the evidence for backwards connections is considered with respect to endogenous connectivity (averaged over experimental conditions) because extant studies have varied this in their model spaces. However, the connectivity of most theoretical relevance is in fact experimental (bilinear) modulation of connectivity. Backwards connectivity that is modulated by experimental factors already accounts for endogenous connectivity and provides stronger evidence that backwards connections are relevant to task-dependent processing. Stronger evidence for the relevance of backwards connections can come also from associations between the magnitude of bilinear parameters and behavior (e.g., face recognition performance).

One phenomenon for which experimental variables modulate backward connectivity is repetition suppression. Repetition suppression occurs when brain areas reduce their functional responses to repeated stimulus information and is commonly used to localize category-specific representations in occipitotemporal cortex (Grill-Spector et al., 2006). As reviewed earlier, more posterior areas (e.g., EBA, OFA) are held to process “lower-level” properties of the category than more anterior areas (e.g., FBA, FFA) (Taylor and Downing, 2011). The conventional explanation for repetition suppression assumes that, as a stimulus is repeated, category-sensitive neurons fatigue and so become less responsive to their preferred input (Grill-Spector et al., 2006). In contrast, predictive coding hypothesizes that higher level areas (e.g., FBA, FFA) can use backwards connections to actively suppress predicted category responses at lower level areas. Because stimulus repetition makes stimulus information increasingly predictable, higher level areas will exert more suppression over lower-level responses.

Empirical evidence was found for this predictive coding hypothesis in two fMRI studies, one examining face-sensitive areas (Ewbank et al., 2011) and the other examining body-sensitive areas (Ewbank et al., 2013). These DCM model comparisons show that repetitions of face or body identity modulate backward connections from fusiform (FBA, FFA) to occipital cortex (EBA, OFA). These DCMs show higher model evidence than fatigue-based DCMs where repetition modulated only local activity, so that repeated information reduced an area’s receptivity to feedforward input. The modulation by repetition on backward connections that observed by Ewbank et al. (2011, 2013) is consistent with predictive coding models where higher hierarchical levels generate predictions which then suppress responses in cells tuned to the predictable information at lower-levels (Friston, 2005), lowering the response to repeated (predictable) stimuli.

Backward connectivity can also be dissociated from feedforward influences by measuring neural oscillations. Directed information transmission between brain areas is inherently linked to neural oscillations and so the study of oscillatory responses complements connectivity analysis. Oscillatory phase relates to coordination between brain areas (Varela et al., 2001; Buzsáki and Draguhn, 2004; Schnitzler and Gross, 2005; Womelsdorf et al., 2007) and routing of information flow (Salinas and Sejnowski, 2001). Distance of information travel may induce oscillation frequencies adapted to different conduction delays (Kopell et al., 2000; von Stein and Sarnthein, 2000). Several studies have identified nested oscillations, or phase-power coupling (Jensen and Colgin, 2007), in relation to directed information flow. And, some varieties of predictive coding models (Chen et al., 2009; Bastos et al., 2012) predict that backward connectivity should involve non-linear signal transformations, expressed as cross-frequency coupling. Chen et al. (2009) used DCM for MEG to test this latter hypothesis for the face perception network by modeling the transfer of frequency-specific power via forward (OFA to FFA) vs. backward (FFA to OFA) connections. Consistent with predictive coding, Chen et al. (2009) observed cross-frequency power coupling only for backward connections (gamma power in FFA suppressed alpha power in OFA) while forward connections showed only same-frequency coupling. They concluded that this pattern of positive linear “driving” forward signals contrasted with non-linear “modulatory” backward signals was consistent with a predictive coding framework (Chen et al., 2009; Bastos et al., 2012). These findings link nonlinear backward influences to viewing of faces, but further research is required to establish a role in behaviorally measured recognition. These results, in sum, show evidence favoring measureable backward influences between occipitotemporal category-sensitive areas. These backward influences may play a role in suppressing responses to repeated (predictable) information. Moreover, they may be non-linear, suppressive and modulatory, properties that are also consistent with predictive coding.

## Backward Influences from Frontal Areas

Frontal cortex has the means for backward modulation of visual cortex via long-range connections. Visual cortex is heavily connected to frontal cortex via the aforementioned fiber bundles: (1) OFC/VMFC via IFOF; (2) OFC/VMFC via the uncinate fasciculus and ILF; and (3) inferior frontal cortex via AF/SLF. Frontal influences on visual cortex have been tested in a number of effective connectivity studies, and these studies suggest that frontal areas can sensitize occipitotemporal visual areas to detect expected stimuli, particularly during visual search.

The more prevalent evidence for this top-down modulation involves OFC or VMPFC (Summerfield et al., 2006; Kveraga et al., 2007; Li et al., 2010; Pantazatos et al., 2012) although some studies implicate more dorsal frontal areas (Mechelli et al., 2004; Liu et al., 2011). Summerfield et al. (2006) show enhanced fMRI responses in VMFC when participants searched for faces (face attentional set) vs. when they searched for houses. Their DCM showed that face attentional set enhances the influence of connections from VMFC to the amygdala and FFA. Pantazatos et al. (2012) used a task that involved searching for an object in a landscape scene and their DCM model comparison showed that this search modulated backward connections from VMFC to an area in lateral occipital cortex (presumably the object-sensitive LOC). They complemented their effective connectivity findings with DTI and visualized the fibers connecting the occipital and frontal areas, fibers which might be related to the IFOF. The idea that frontal cortex sensitizes visual areas to detect expected stimuli gains further support from two studies that used DCM for fMRI to show that frontal modulation of visual cortex responses are associated with illusory perception when participants search for (non-existant) letters or faces in noise. In one of these studies (Liu et al., 2011), DCM model comparison showed that illusory letter detection modulated connections from a dorsal area in frontal cortex to left middle occipital gyrus (presumably the visual word form system). In the other study (Li et al., 2010), DCM model comparison showed that illusory face perception modulated reciprocal connections between OFC and OFA. Kveraga et al. (2007) provide more specific data on what information OFC might be transmitting to occipitotemporal cortex. Their study used DCM for fMRI to show that the backward connections from OFC to lateral occipital cortex (presumably LOC) are modulated by stimuli that preferentially stimulate the magnocellular channel of the visual system, suggesting relatively coarse, low-spatial frequency visual information.

Frontal top-down modulation of visual cortex during attempted detection of stimuli involves the use of visual information from memory and so frontal cortex could be involved in other tasks where visual memory has top-down influences on visual responses. Indeed, when participants imagined (from memory) faces, chairs or houses during fMRI scanning, significant category-specific bilinear parameters were observed on connections from prefrontal cortex to face-, chair- and house-sensitive ventral temporal areas (Mechelli et al., 2004). However, when participants viewed these three categories without demands to retrieve their appearance from memory, significant category-specific bilinear parameters were instead observed on forward connections from low-level occipital cortex to the three category-sensitive ventral temporal areas (Mechelli et al., 2003, 2004). This dissociation between imagery-driven top-down vs. stimulus-driven bottom-up modulation reinforces the proposal that frontal cortex guides visual responses based on top-down information in memory.

## Backward Modulation from Amygdala is Associated with Emotional Processing

Backward modulation of higher-level visual areas by areas outside of visual cortex may also occur when processing emotional visual material, such as expressive faces. Enhanced responses to fearful expressions and threatening or aversive scenes have been observed in the fusiform gyrus and other occipitotemporal areas (Sabatinelli et al., 2011). These emotion-related responses are hypothesized to come about via feedback from the amygdala (Morris et al., 1998; Vuilleumier and Pourtois, 2007; Sabatinelli et al., 2011). An early DCM model comparison addressed whether feedforward or backward influences associated with FFA or STS gives rise to occipitotemporal emotion-related responses (Fairhall and Ishai, 2007). This study found that fearful facial expressions modulated feedforward connections from OFA to amygdala, via the FFA. Other effective connectivity studies yielded limited conclusions with respect to the amygdala because they (1) did not include direct connections modulated by threat or fear from the amygdala to occipitotemporal areas in their model comparison space (Dima et al., 2011; Goulden et al., 2012); or (2) tested for endogenous coupling between amygdala and occipitotemporal cortex rather than emotion-modulated (bilinear) coupling (Rudrauf et al., 2008; Herrington et al., 2011); or (3) used EEG (Rudrauf et al., 2008; Keil et al., 2009) or MEG (Furl et al., 2014a), which may not be sensitive to amygdala responses (Attal et al., 2012).

More direct effective connectivity evidence favoring amygdala feedback comes from Furl et al. (2013) who used DCM for fMRI and a more comprehensive model space (over 500 models) than Fairhall and Ishai (2007). Furl et al. (2013) found that fearful expressions evoked enhanced responses in fusiform gyrus only for static faces, while fearful expressions evoked enhanced responses in hMT+/V5 and posterior STS instead for dynamic faces. Their DCM model comparison showed that both the responses in FFA (to static fearful faces) and responses in hMT+/V5 and STS (to dynamic fearful faces) could be accounted for by amygdala feedback. The results of Furl et al. (2013) therefore not only favor an amygdala feedback account, but they also suggest an active role for the amygdala in top-down control over motion and form processing.

Another prominent hypothesis regarding the amygdala supposes that it initially receives and processes expedited visual input via short cut pathways, which enable the amygdala to modulate visual cortex more quickly (Morris et al., 1999). The existence, anatomy and number of such pathways to the amygdala remain controversial, with some studies claiming that the amygdala receives visual input via the cortical dorsal stream (which may involve the motion-sensitive hMT+/V5 and parietal areas) and other studies instead proposing a subcortical route from the retina via the pulvinar and superior colliculus (Vuilleumier and Pourtois, 2007; Pessoa and Adolphs, 2010). Anatomic evidence in the monkey for this subcortical visual pathway from the retina to the amygdala has not been forthcoming. For DTI data in the human, Tamietto et al. (2012) showed a tract that could potentially form part of this pathway. Although this study showed connections between superior colliculus, pulvinar and the amygdala, the direction of information transfer cannot be determined using DTI. Furl et al. (2013) included in their model comparison DCMs with an additional face input to the amygdala but found better evidence favoring models without this input, suggesting a cortical route to the amygdala. Rudrauf et al. (2008) used DCM to model EEG responses to pictures of objects and demonstrated a pathway to an anterior temporal area potentially encompassing the amygdala from either V1 or the thalamus (these two options produced equivalent model evidence).

Thus, despite* a priori* hypotheses about pathways to amygdala, effective connectivity and DTI evidence has not so far not revealed any particular pathway. Effective connectivity studies remain to be performed using conditions thought to favor the usage of a subcortical pathway: when faces are low spatial frequency, peripherally or subliminally presented or unattended (Morris et al., 1999; Anderson et al., 2003; Winston et al., 2003; Williams et al., 2004). Effective connectivity studies would also be complemented by DTI in this case, as the major fiber bundles may play roles both in conveying visual information to the amygdala as well as conveying its modulatory feedback to visual cortex. The ILF, for example, may directly connect ventral occipitotemporal areas with amygdala. It has also been suggested that the dorsal pathway could convey coarse and fast magnocellular visual information to the amygdala (Pessoa and Adolphs, 2010), implicating the more dorsal arcuate fasciculus. The amygdala could also connect with posterior visual areas via the OFC through the IFOF and uncinate fasciculus.

## Conclusions and Future Directions

This article reviews recent imaging studies of human category-sensitive visual areas with a view to describing their responses in terms of effective influences from structurally-connected areas. The majority of this work involves face perception and, in the case of faces, our conclusions agree with a recent review of lesion and TMS data (Atkinson and Adolphs, 2011), which also emphasizes roles for backward connections and areas that are not face-sensitive in the visual analysis of faces. Although a feedforward hierarchy comprised of only face-sensitive areas appears to be a straw-man perspective, the prevailing account of the visual analysis of faces (Haxby et al., 2000) does not go substantively beyond it and many computational models of high-level vision emphasize only feedforward interactions (e.g., Riesenhuber and Poggio, 1999; Giese and Poggio, 2003). Structural and effective connectivity evidence suggests that visual (core) analysis of faces is not limited to face-sensitive occipitotemporal areas (OFA, FFA, posterior STS) but includes other areas within occipitotemporal cortex. For example, FFA is structurally connected to areas which are not face-sensitive, including LOC and hMT+/V5. Beyond visual cortex, the amygdala, OFC/VMFC and inferior frontal cortex might exert directed effective top-down influence on responses in category-sensitive occipitotemporal areas, perhaps via large fiber bundles such as ILF, IFOF and AF/SLF.

## Open Questions

This reviewed research poses several new questions that may be addressed using a functional integration approach. (1) Effective connectivity analyses should be combined with DTI to investigate the functional specialization of the major fiber bundles (e.g., IFOF, ILF, AF/SLF). (2) These fiber bundles may support fast feedforward shortcut pathways that trigger early influences of backward connections (Lamme and Roelfsema, 2000; Pessoa and Adolphs, 2010; Panichello et al., 2013). In the case of putative shortcut pathways to the amygdala, controversy remains over whether such shortcuts are cortical or subcortical (Pessoa and Adolphs, 2010). Effective connectivity analyses could be combined with DTI to identify anatomy and function of these shortcut pathways. (3) ILF and AF/SLF are associated with both long fibers and shorter U-shaped fibers that can respectively contribute to shortcut connectivity and communication between adjacent hierarchical levels (Tusa and Ungerleider, 1985; ffytche and Catani, 2005). Existing studies examining contributions of fiber bundles have not separately examined these types of fibers (e.g., Thomas et al., 2008, 2009) and their roles should be dissociated. (4) Responses to different categories (faces, bodies, objects, biological motion, etc.) are typically studied in separate experiments. The evidence reviewed here favors structural connectivity between areas sensitive to different categories such as LOC, FFA and hMT+/V5. More work should examine how object, place, word, body, face and biological motion areas structurally and effectively interconnect. (5) As the network of areas known to be connected together in support of a function gets larger, metrics such as graph theory will become be more important for understanding how network organization predicts function (Sporns and Zwi, 2004). (6) The face-sensitive area in posterior STS shows poor structural connectivity with its fellow face-sensitive areas (Blank et al., 2011; Ethofer et al., 2011; Gschwind et al., 2012; Pyles et al., 2013). The structural connections between the face-sensitive area in posterior STS and other face-sensitive areas should be investigated further. Does hMT+/V5 mediate this communication? (7) Under some circumstances, the feedforward sweep appears sufficient to engender stimulus-dependent neural responses (Lamme and Roelfsema, 2000), including high-level category sensitivity (Oram and Perrett, 1992; Tovée, 1994; Liu et al., 2009). And, some connectivity evidence favors feedforward networks in visual cortex over reciprocally-connected ones (Fairhall and Ishai, 2007; Furl et al., 2014a). However, structural backward connections are prevalent in cortex and there is substantial evidence for backward influences among occipitotemporal visual areas (Rudrauf et al., 2008; Ewbank et al., 2011, 2013; Liu et al., 2011; Goulden et al., 2012; Nagy et al., 2012). The circumstances under which the influence of backward connections are measureable need to be determined. (8) Effective connectivity studies have examined backward modulation by spatial attention for low-level stimuli (Friston et al., 2003). Less is known for higher-level functionally-defined areas. Moreover, backward influences on spatial attention from parietal cortex have not been be dissociated from other forms of backward influence. For example, frontal cortices appear to predict or search for stimuli and the amygdala may orient attention to emotionally-relevant stimuli. How do these types of attentional orientation relate to parietal spatial attention? (9) How can the emerging roles for backward and other influences be accommodated in computational models, given that some of the more commonly-used models are solely feedforward? One modeling framework which has recently received attention is predictive coding. Predictive coding frameworks assign clear roles for forward and backward influences and provide a flexible framework for developing testable models (Friston, 2005; Bastos et al., 2012).

Some of the new research questions raised here concern fundamental mechanisms that may be repeated throughout the brain and involved in multiple functions beyond high-level vision. One example involves the role of backward connections. The computational advantages of implementing backward connections over using purely feedforward models (as in, for example, predictive coding) may be useful for understanding computations outside the visual system. Oscillations are another widespread phenomenon which can be investigated using connectivity-based methods and have been proposed to be a fundamental mechanism for brain communication (Salinas and Sejnowski, 2001; Varela et al., 2001; Fries, 2009). The effective connectivity methods reviewed here for measuring frequency-specific power coupling have already been applied to functional imaging of the motor system (Chen et al., 2010, 2012). Another phenomenon that is prevalent outside of higher-level vision is repetition suppression (i.e., neuronal adaptation). The effective connectivity studies reviewed here (Ewbank et al., 2011, 2013) suggest a mechanism—top-down suppression from higher-level areas—which may operate for lower-level vision or other modalities as well.

## Limitations of Existing Studies

Although connectivity-related methods such as DTI and effective connectivity can potentially address the questions posed above, it is important to be realistic about limitations imposed by these methods. DTI and effective connectivity share the same disadvantages inherent in brain imaging in general and so inferences should be informed and/or validated by animal lesion, anatomy and neurophysiology research. DTI and effective connectivity analyses have their own weaknesses as well. DTI, for example, has limited utility for distinguishing the direction of connections (e.g., forward vs. backward). Effective connectivity analysis cannot determine whether a connection between two areas is direct or indirect. Methods such as DTI, effective connectivity and conventional functional imaging methods based on the general linear model can overcome their weaknesses when used in conjunction.

Effective connectivity analysis applied to fMRI data can also be problematic, due to the uninformative temporal resolution of the blood-deoxygenation response signal. This problem is acute for methods such as Granger Causality and structural equation modeling, which depend on exact timing of fMRI response signals to draw inferences about connectivity between underlying neuronal sources (Penny et al., 2004b; Friston et al., 2013). DCM addresses this timing issue by employing a mapping between neuronal sources and the measured fMRI response. Many of the estimable parameters in DCM concern details of this mapping (Friston et al., 2003). Nevertheless, as the complexities underlying the generation of the fMRI signal from neural responses is better understood (Aquino et al., 2012, 2014), the model of this mapping will need to be updated accordingly. At present, DCM for fMRI has received some direct validation from concurrent neural recording in rodents (David et al., 2008). Hemodynamic model development can also be assisted by DCM model comparison, which provides a method for identifying which competing model of hemodynamics best predicts fMRI data (Stephan et al., 2007). More work is needed comparing DCM for fMRI against data from MEG, EEG and local field potentials, which directly measure post-synaptic potentials and provide a richer source of neuronal dynamics for modeling. Moreover, computational simulation work would reveal the robustness of DCM to hemodynamic phenomena not accounted for by its current hemodynamic model (Aquino et al., 2012, 2014).

A particularly vexing challenge in applying* a priori* model based methods such as DCM is specifying a model space that is (1) computationally tractable; (2) includes all plausible models; and (3) can be adequately compared to model spaces used in similar studies. The present review presents the outcomes of model comparisons, even though the model spaces used by each study vary widely, often omit plausible models that are of theoretical interest or estimate the parameters of only one model. Newer methods such as *post hoc* model comparison (Rosa et al., 2012) and model family comparison (Penny et al., 2010) can help manage populous model spaces. A key strategy for managing model spaces is to use empirically observed univariate effects of experimental conditions to guide decisions about bilinear (condition-specific) modulation. For example, it is not plausible for a connection to be modulated by faces vs. non-faces if its target brain area does not respond more to faces than non-faces. Many studies reviewed here, particularly early ones, do not report all of their univariate effects and do not use them to constrain their model spaces (e.g., Fairhall and Ishai, 2007). Thus, some model spaces are contaminated by implausible models, including models that are not appropriate to explain observed univariate effects. Furl et al. (2014b) provides a recent example of a study where the model space is specifically designed to explain a univariate effect, in this case, an interaction of motion (dynamic vs. static) and form (face vs. non-face) where the STS responds strongly only to dynamic faces. Out of the different possible ways that this univariate effect could have been produced via network actions, Furl et al. (2014b) shows that face-sensitive activity in OFA (but not FFA) gated, or modulated, motion information transmitted from hMT+/V5. This finding reveals that areas sensitive to motion and form interact when influencing STS, with the form pathway acting as gain control on the motion pathway. The placement of bilinear and non-linear connections in this model space was motivated by the areas which showed univariate main effects and interactions of form and motion and resulted in both a reasonably-sized space of plausible models and a network-based explanation for these univariate effects.

## Conclusion

This article reviews recent work examining structural and effective connectivity associated with category-sensitive areas in high-level visual cortex in the human. These studies add to our knowledge of the functions of forward and backward interactions between visual areas and top-down influences from frontal areas and the amygdala. More work is needed to develop theory that moves beyond a focus on the modular nature of individual areas with only feedforward interactions. Structural and effective connectivity studies have not yet given us a complete picture of network interactions and, like any method, possess both strengths and limitations. Nevertheless, they allow direct testing of connectivity in ways that complement more conventional uses of brain imaging and therefore hold promise for answering numerous future research questions.

## Conflict of Interest Statement

The author declares that the research was conducted in the absence of any commercial or financial relationships that could be construed as a potential conflict of interest.
